# The current and potential role of community pharmacy in asset-based approaches to health and wellbeing: a qualitative study

**DOI:** 10.1007/s11096-021-01244-z

**Published:** 2021-02-26

**Authors:** Jayne Astbury, Ellen Schafheutle, Jane Brown, Christopher Cutts

**Affiliations:** 1 Division of Pharmacy and Optometry, Faculty of Biology, Medicine and Health, Centre for Pharmacy Workforce Studies, School of Health Sciences, The University of Manchester, Room 1.136, Manchester, M13 9PT UK; 2 Health Education England, Manchester, UK

**Keywords:** Asset-based approaches, Community pharmacy, Integration, Public health

## Abstract

*Background* Asset-based approaches seek to positively mobilise the strengths, capabilities, and resources of individuals and communities. To date, limited consideration has been given to the potential value of this approach in relation to community pharmacy practice, yet this is important and timely given community pharmacy’s expanding role and contribution to public health initiatives. *Objectives* This qualitative study aimed to explore the current and potential role of community pharmacy in asset-based approaches. *Methods* Fifteen semi-structured telephone interviews were undertaken with community pharmacists and project leads, and public health policy and strategic leads in the UK. Transcripts were analysed using simultaneous inductive open and deductive coding using an applied Theory of Change as an illustrative lens. *Results* The shift towards patient-facing roles in community pharmacy was felt to offer expanded relational opportunities to engage and collaborate with individuals, communities, and other stakeholders. However, only a small number of respondents described examples of systemic asset-based working within the pharmacy sector. The adoption of asset-based approaches was challenged or enabled by several factors including the availability of protected time/resources, workplace and organisational culture/values, strategic leadership, commissioning, and funding arrangements. *Conclusions* The study provides valuable insights into the potential for community pharmacy, a previously unconsidered sector, to further adopt and contribute to asset-based approaches and play a more central role in the improvement of public health and reduction of health inequalities.

## Impact of findings on practice

- There is scope for community pharmacy teams to incorporate and contribute to asset-based approaches in their localities.
- The further adoption of asset-based approaches requires a programme of enabling funding, strategic leadership, and changes in workplace culture.

## Introduction

As in other countries, United Kingdom (UK) local authorities and National Health Service (NHS) organisations face significant challenges in supporting population and public health in the context of limited resources, an ageing population, increasing prevalence of long-term conditions, and widening health inequalities. Asset-based approaches, often considered and positioned as a critical counterpoint to traditional deficit-based approaches, offer an alternative way of working with individuals and communities [1]. Asset-based approaches aim to improve wellbeing, protect against ill-health, and reduce health inequalities by mobilising, capitalising, and sustainably building upon the existing capacities, strengths, relational networks, and resources of individuals, families, and communities [2].

Asset-based approaches are increasingly being adopted as a foundational principle within UK health and social care policy and legislation [3, 4], used as a framework to support the integration of health and social care services, and to underpin the reconfiguration of funding arrangements in order to meet place-based health priorities [5]. Community pharmacies represent a socially inclusive health resource, with approximately 50% of the UK population living within 500 m of a pharmacy [6]. Besides medicines supply and medicines/clinical services, community pharmacy practice also occupies an increasing role within public health and prevention [7], predominantly delivered through the Healthy Living Pharmacies (HLP) framework [8].

The HLP framework aims to support pharmacies to proactively promote and influence health and wellbeing in their locality. Health champions play a key role and provide a range of public health interventions in addition to offering self-care advice and signposting to local resources. Health champions are commonly not pharmacists but support staff, such as pharmacy technicians, dispensing assistants, medicines counter assistants, and delivery drivers who have significant face-to-face contact with people accessing pharmacy services [9]. Considering that community pharmacies are established providers of public health services, scant consideration has been given to their role within asset-based approaches and their ability to engage with and contribute to the health and wellbeing of the communities in which they are situated.

## Aim and research objectives

This study aimed to explore the current and potential role of community pharmacy in asset-based approaches and sought to address the following research objectives: To investigate if and how community pharmacists have adopted asset-based approaches into their practice, and to explore the views of community pharmacists and other stakeholders with regards to the value and potential scope of asset-based practice involving community pharmacy.

## Methods

### Ethics approval

This study received ethics approval from The University of Manchester [2019-6796-10316].

### Theoretical framework

The study adopted the Theory of Change for Asset-Based Approaches (TCABA) [10] as a theoretical framework. Theory of Change considers the ‘central process or drivers by which change comes about for individuals, groups or communities’ [11], and provides a comprehensive methodology with which to map out long-term outcomes of change initiatives within particular contexts. The TCABA posits four key iterative stages for the adoption of asset-based approaches within local systems and as such provides a valuable lens through which to consider diverse levels of integration in a variety of locality settings. The four stages include reframing towards assets, recognition of assets, mobilising assets, and co-production.

### Sample and data collection

Sampling was purposeful [12] and included two main groups of participants from across the UK: the first group was comprised of community pharmacy practitioners and pharmacy project leads, which included representatives of professional pharmacy bodies; the second group included public health policy and strategic leads. An approximate target sample size was estimated at 12–15 interviews [13]. The final sample size of 15 was informed by regular evaluation of data saturation [14]. Participants were recruited using email invitation and recruitment advertisements cascaded via professional networks and social media. Inclusion criteria mandated previous or current involvement, or professional interest in, asset-based initiatives or innovative public health programmes.

The four stages of the TCABA informed the development of a semi-structured interview schedule which included open ended questions and prompts to explore how assets and asset-based approaches were understood, identified, and assimilated into practice and systems, and the perceived barriers and facilitators to successful implementation (see Table 1). The research team included a pharmacy workforce and policy researcher (ES), two post-registration pharmacy educators (CC and JB), and a health services researcher with a background in community nursing and an interest in public health (JA). All were independent of service innovation and design. Reflexivity was encouraged at all stages of the research through regular discussions concerning positioning and interpretation [15]. Following university ethics approval and with written/verbal consent, telephone interviews (enabling national coverage) were conducted by JA, digitally recorded, transcribed verbatim, and anonymised.

**Table 1:** The four stages of the TCABA and related interview questions

TCABA Stage	Interview questions
Stage 1: reframing towards assets	When you use the term asset-based approaches what are you referring to? Can you explain what you mean by asset-based approaches? Can you give any examples?
	Have you or your colleagues received any training on asset-based approaches? Do you feel that this approach is widely understood within your team/organisation/locality? Please explain
	Are there key individuals championing asset-based approaches in your team/organisation/locality? How do they do this?
Stage 2: recognising assets	Can you explain what you mean by the term ‘assets’ in the context of this approach?
	How do you/people in your team/organisation/locality become aware of/identify community assets?
	What role do you feel community pharmacies/pharmacies could have in terms of this approach? What do you feel are the possibilities with regards to community pharmacies/pharmacists in terms of adopting asset-based practices and/or becoming involved in asset-based approaches in their localities?
Stage 3: mobilising assets	Can you give me examples of asset-based approaches within your team/locality/organisation?
	Are you aware of any examples of asset-based approaches involving community pharmacies/pharmacists?
	What would need to happen/be in place for community pharmacies/pharmacists to adopt this way of working into their practice and/or become involved in asset-based approaches in their localities?
	What might be the barriers/challenges?
Stage 4: co-producing assets and outcomes	How do you/your team/organisation identify/evaluate intended outcomes of this approach to working with individuals or communities?
	Has this involved any collaboration/consultation with patients/customers/citizens, communities, or organisations? Please explain

### Data analysis

Data analysis was undertaken by JA supported by the software NVivo^14^. Transcripts were analysed using a hybrid approach of simultaneous inductive open coding, and deductive coding utilising a framework of a priori codes drawn from the TCABA. This interpretive approach allowed the theoretical framework to be applied whilst allowing additional themes to emerge from the data [16]. The coding frame was developed and discussed iteratively at regular meetings to critique interpretation and reduce bias [17]. Differences in interpretation were resolved by revisiting the data and discussing the coding until consensus was reached. A number of subthemes emerged that were highly specific to the commissioning of community pharmacy services, e.g. Healthy Living Pharmacies. No new overarching themes were identified outside those depicted within the TCABA.

## Results

Fifteen participants were interviewed: eight public health policy and strategy leads (policy), including public health, mental health, local authority, and voluntary sector roles; and seven community pharmacists or pharmacy project leads/representatives (pharmacy). Interviews ranged from 25 to 60 min in duration. The findings are presented under the four iterative stages of the TCABA.

### Stage 1: reframing towards assets

The TCABA suggests that effective implementation rests upon a shared understanding of asset-based concepts and theory, and the reframing of resources and priorities towards asset-based values and practice. There was broad consensus amongst participants with regards to the meaning they attributed towards the term ‘asset-based approach’ which was typically depicted as a place-based approach that aims to recognise and build upon the existing strengths of individuals and local communities. This strengths-based focus was frequently framed as an intentional divergence away from traditional deficit-based models of service provision:

Asset-based working is working with local people in a different way that seeks to recognise and nurture the strengths of individuals, families, and communities and to help them build independence and self-reliance (Interview 10—Policy).

Pharmacy participants largely conceptualised their current and potential involvement in asset-based approaches at the level of working with individuals or communities. On an individual level, asset-based approaches were understood as the adoption of increasingly person-centred and strength-based approaches towards consultations with patients/customers:

Instead of looking at somebody’s weaknesses and things that they, maybe, don’t do so well, it’s having a conversation to find out where their strengths are….What is it that they enjoy doing, what is it that matters to them in life, not what I think matters but what they think matters (Interview 6—Pharmacy).

At a community level, pharmacy interviewees saw asset-based working as the ‘contribution’ that community pharmacies made, or could make, to the communities in which they were situated beyond current commissioned pharmacy services. Policy and strategic leads additionally conceptualised asset-based approaches on a more systemic level, as a strengths-based philosophy that could be utilised to inform and underpin health and social care service design, structure, and delivery:

The overarching use of the term to me is to describe a world view where health and social care services are not done to people, where they are seen as ill and needing benevolent help, which is really a passive model. But more whereby we recognise what exists in the community, the assets that are there, and we have structures, and processes, and ways of working to harness those to support wider health and well-being (Interview 14—Policy).

The need for increasingly collaborative relationships between individuals and communities, and health and social care services, local authorities, and policy makers was highlighted. Arrangements facilitating shared decision-making and devolvement of power were seen to be key in terms of meeting local priorities and empowering citizens and communities to have greater control and authority with issues concerning their health, lives, and local area.

I think the nature of asset-based approaches is much more about shifting power into those communities and letting them and the organisations and groups that are there, determine what the best model of support looks like, that fits the needs of local people (Interview 14—Policy).

The rebalancing of power towards a more equitable relational dynamic was also referenced with regards to interactions between citizens and health professionals. All participants acknowledged that this shift in perspective and practice required a corresponding change in professional identity and culture, and that this reframing could conflict with existing professional mind-sets:

The big challenges tend to be cultural for the healthcare professionals. To move away from this mind-set that we are here to cure people, or if we can’t cure them, because it’s a long-term condition, we are here to provide that support and advice. So, it disempowers people to self-care more effectively. So, it’s that mind-set which says, this is down to an individual themselves, our role is to help them access the support that’s around them, and to think of that support much more broadly than clinical support (Interview 14—Pharmacy).

The language used by some pharmacy participants when discussing their adoption of asset-based approaches, suggested that there was a tendency to continue to frame their involvement with customers/patients from the standpoint of their role as ‘expert’. Interactions were commonly characterised as unidirectional ‘advice giving’ and professional-led ‘interventions’ as opposed to person-centred or strengths-led, suggesting a lack of understanding or coherence in the adoption and integration of asset-based values into practice:

I think community pharmacists, they just do things and they just help, they just make these interventions, and they just help people out, and they just fix these problems (Interview 15—Pharmacy).

### Stage 2: recognising assets—the potential of community pharmacy

Identifying and recognising existing assets, including an awareness of how they can be accessed, utilised, and connected is key to mobilising and capitalising upon the resources available to individuals and communities. Participants typically conceptualised ‘assets’ as existing resources or capacities present within individuals, organisations, or communities that created or contributed to health and wellbeing. The majority of policy leads suggested that there was significant scope and potential for community pharmacies and their workforce to be further recognised and utilised as health assets within their communities. The rationale and value of more effectively incorporating community pharmacy into asset-based approaches centred upon accessibility:

Community pharmacy is probably the only remnant of the NHS that sits on the high street, that is part of the community, and people can go into that space not necessarily to receive an NHS service, they go into community pharmacies for all sorts of reasons […] and […] it is pretty much the only place you can walk into and, in a very informal way, have a conversation with a healthcare professional (Interview 1—Policy).

The very established physical presence of community pharmacies within local communities was seen to provide a tangible opportunity for them to act as assets within their neighbourhoods. Community pharmacies were seen to occupy a unique position in terms of the broad range of people accessing their services and the scope to undertake proactive public health work:

The other advantage of community pharmacy is that the staff are far more likely to encounter the otherwise well people who wouldn’t necessarily access other health services. So, there is great opportunity to work proactively, and even to go out and work with other organisations as well” (Interview 9—Pharmacy).

Community pharmacy’s drive towards more patient-facing services, including public health initiatives, was seen as an opportunity to further develop relationships with individuals and communities. The potential for pharmacists to expand their social value and contribution to social capital was also referenced by several policy and strategy leads. The notion of social value centred on creating and building connections with other local agencies and resources:

The thing that I am really interested in is, [community pharmacy] expanding their social value at a kind of community level…. Local pharmacies are like other businesses, but they have a particular part to play in what’s going on in a locality, have the connection to people who have long term needs, that few other places within a local community have” (Interview 12—Policy).

Building on community pharmacies’ current involvement in public health and prevention was seen as a potential avenue to develop their patient-centred and asset-based roles. Policy and strategy leads highlighted the potential to better utilise the existing resources and skills within the pharmacy workforce, with emphasis on workers other than pharmacists, such as health champions:

There are 9500 health champions in community pharmacies …they’re not a registered profession, they’re not pharmacists, they’re coming from the local community and they’re seen as members of that community, in touch with the community. They are going out there doing health related interventions, so I could see that there is potential there (Interview 3—Policy).

The potential to develop and build upon existing relationships, and repeated contact with patients/ customers, particularly those receiving repeat prescriptions, was also noted. Improved access to, and inclusion, in asset directories/mapping initiatives and resources was seen as fundamental to enabling the development of a reciprocal and dynamic awareness between pharmacy teams, other health and social care services/sectors, as assets within the community. It was thought that this would support more effective signposting from and to pharmacies.

### Stage 3: mobilising assets—current examples of asset-based practice

Mobilising assets involves putting the assets that have been identified to work by catalysing active connections and networks between assets, individuals, and communities. Only one example of systemic implementation of asset-based approaches in community pharmacy emerged during the interviews: ‘Building the Community Pharmacy Partnership’ (BCPP) project (https://www.cdhn.org/bcpp), a grant programme funded by the Health and Social Care Board in Northern Ireland that aims to support community pharmacies to work in partnership with local communities to address locally identified and defined needs. Other examples of asset-based practice tended to relate to ways in which pharmacy teams had adopted strengths-based approaches to consultations with patients/customers, as well as the practice of signposting individuals to community resources:

Really taking advantage of the assets that a particular community may have, whether that’s a leisure centre, whether that’s a support group. It ties in quite closely with signposting from pharmacies, one of our aims is to make sure that [pharmacy staff] are aware of all of the available services, assets within an area, so that they are able to signpost people more effectively (Interview 9—Pharmacy).

Community pharmacists provided a few discreet examples of asset-based approaches, including setting up and supporting group sessions and activities, engaging with voluntary sector projects, and applying for funding in conjunction with other organisations.

We [the pharmacy] trained one of our counter staff to become a health leader… we set up a walk every Thursday with the idea of…well the walk's healthy and that's a good thing, that was kind of the excuse, but actually it's more about …could these people set up a new kind of social network. Then we had to make that sustainable, so we managed to get one of them trained up to be the lead. Now they just meet…it's still going 15, 16 years later. They just meet in the pharmacy every Thursday. (Interview 8—Pharmacy).

The need for greater strategic direction and proactive leadership within the community pharmacy sector was noted. Some policy leads highlighted the absence of pharmacists in relevant regional and specialist networks*.* The need for proactive strategic representation of pharmacy in policy and decision-making forums was stressed so as to ensure community pharmacy was considered and incorporated in discussions concerning local commissioning. Funding was depicted as a key enabler in facilitating the adoption of new ways of working, particularly when this involved time away from the pharmacy premises. Policy and strategic leads highlighted the tension inherent in developing approaches to commissioning that operated at scale whilst also retaining local sensitivity:

The approach is not about having a standard approach everywhere, one size fits all, it’s about systematising this way of working. So that is, each locality deciding themselves which approach to use, is it local area coordinators, is it social prescribing, is it integrated pharmacy services, is it pharmacy health champions. Is it a combination of all of those? (Interview 3—Policy).

#### The role of healthy living pharmacies (HLP)

The HLP framework was suggested as a potential mechanism by which to provide enabling funding, begin to embed asset-based approaches into community pharmacy practice, and further mobilise their public health contribution. Participants raised the possibility of further development of the HLP framework to include a component or level concerning community pharmacies’ engagement with and contribution to their local community, broadly aligned to the notion of social capital. The recent introduction of the HLP concept was seen as a largely positive step towards establishing a framework for pharmacies to consider and establish locally sensitive services focused upon public health and wellbeing. However, several pharmacy participants highlighted limitations related to the current reimbursement model. The shift to commissioning Level 1 HLP services via quality payments was perceived to have had a detrimental impact on service quality, as the level of minimum standards was seen to enable tokenistic service provision as opposed to promoting genuine engagement and culture change. Pharmacists highlighted the concurrent tension between the role of standardised regulation and benchmarks and the provision of quality, locally sensitive, and innovative services:

A way to guarantee that people engage with these things is to include them in say, quality payment. But does that necessarily mean that you will get a good service from all those pharmacies or will they just do the bare minimum to get what they need? So, it does involve regulation as well (Interview 9—Pharmacy).

### Stage 4: co-production—creating an enabling climate for change

In terms of generating additional opportunities for the adoption of asset-based practices, local service delivery networks were seen as a potential way of enabling community pharmacists to become more linked in and able to contribute to existing community services and resources. Policy leads cautioned against the community pharmacy sector duplicating work already being undertaken by other groups and services. It was felt that the proactive development of collaborative relationships with other organisations and sectors skilled in community consultations and engagement would enable community pharmacies to understand how they could most effectively contribute to their local community, integrate into existing asset-based systems, and collaborate in emerging initiatives, such as social prescribing. Supporting community pharmacists to forge those relational connections was seen as key to enabling further development:

If I was doing development work with pharmacists then I’d be tempted to bring together a group of community pharmacists who want to get more involved with their local community and do a series of workshops that explored how to do it in a practical way, building on their own strengths, knowledge, data, and possibly putting some money alongside it. (Interview 12—Policy).

## Discussion

This qualitative study adopted the TCABA as a theoretical lens to explore the current and potential role of community pharmacy within asset-based approaches to health and wellbeing. The findings add to the community pharmacy and public health literature by qualitatively describing the degree to which asset-based approaches have been understood and incorporated into community pharmacy practice, and the barriers and enablers to implementation. Opportunities for the expansion of asset-based practice within community pharmacies are also highlighted.

There was evidence of an emerging shift towards asset-based thinking and values amongst pharmacy participants who typically conceptualised asset-based approaches at the level of working with individuals and communities. On an individual level, asset-based approaches were understood as the adoption of increasingly person-centred and strength-based approaches towards consultations with patients/customers. However, the language used by some pharmacy participants indicated that there was a tendency to persist in framing their relationships and interactions with customers/patients from the standpoint of professional ‘expert’. Previous research suggests that community pharmacists have limited understanding of person-centred conversations and care planning [14], and tend towards medicine-focused, rather than person-centred consultation styles [18]. Moreover, some pharmacists have been found to lack confidence in undertaking consultations concerning patient-led goals and healthy lifestyle [19], and feel uncomfortable not using their medicines expertise [20]. Rippon and South [10] note that adoption of asset-based values can often conflict with professional identity and organisational ideals and that meaningful implementation of asset-based practice requires a shift in practice culture and corresponding change in language. The lack of coherence and consistency with which asset-based practice was understood and described in this study suggests that there is a need for further delineation and operationalisation of asset-based terminology across the community pharmacy sector. The TCABA highlights the role of ‘champions’ and supportive leadership in the process of reframing towards asset-based practice. Participants highlighted an absence of strategic leadership within the community pharmacy sector and a lack of pharmacy representation within relevant regional and specialist networks. The development of proactive leadership roles is necessary to enable the sharing of innovative practice and the forging of collaborative relationships with other professions, sectors, and businesses.

At a community level, asset-based working was seen as the ‘contribution’ that pharmacies made, or could make, to the communities in which they were situated beyond current commissioned services. This contribution was often aligned with the notion of strengthening social capital and non-medicalised approaches to enhancing individual and public wellbeing, including emerging initiatives such as social prescribing [21]. The role of community pharmacists in relation to social capital and community development has been previously considered by Ghalamkari and Jenkins [22] and Bissell [23]. Both authors highlight that community pharmacy’s positioning as a local resource, provider of NHS services, and commercial business, places the sector in a unique position to generate and add social value. Bissell [23] noted barriers including a lack of remuneration and attitudinal reticence with regards to roles aside of medicines expertise. Participants within the current study suggested that consideration of and contribution to social capital was an activity that could potentially be evaluated and remunerated as a means of enabling pharmacies to become more involved in community development activities. Given the now more established nature of public health work within community pharmacy it is perhaps timely to re-explore and re-evaluate the scope for pharmacy to add increased social capital and value to the communities in which they are situated [24].

With the exception of the BCPP programme, there were few examples pertaining to the systemic adoption of asset-based working within the pharmacy sector. The potential value of further developing and integrating asset-based approaches within the context of community pharmacy centred upon their established physical presence and accessibility. This is supported by evidence indicating that community pharmacy is the sole exception to the inverse care law described by Tudor Hart [25], with the prevalence of community pharmacies being greatest in areas of higher social deprivation [26]. The changing landscape of community pharmacy practice and shift towards patient-facing clinical roles was felt to offer expanded relational opportunities for pharmacists and the wider pharmacy workforce to engage and collaborate with individuals, communities, and other stakeholders.

The lack of systemic funding was cited as a key barrier to further adoption and implementation. Enabling community pharmacy to adopt asset-based practices will require the development of a funding model that can accommodate the necessary paradigm shift [27]. Participants perceived the development of the HLP concept as offering a potential mechanism to incentivise and reimburse asset-based working in community pharmacy. The development of resources, support, and guidance regarding community engagement and the development collaborative relationships with other services and sectors is also warranted. Although grounded within the context of UK healthcare and public health service provision, the findings offer insights with respect of further utilising the skills and strengths of the community pharmacy organisations and their workforce in engaging and working collaboratively with communities to tackle the wider social determinants of health inequalities, drawing upon and developing existing service programs as a potential mechanism for reimbursement and incentivisation.

## Limitations

The small-scale nature of the study and small sample size must be acknowledged as limitations. Furthermore, whilst the TCABA was chosen as a framework with which to interpret the study findings, this model is at relatively early stage in development and is therefore intended to be illustrative. The absence of consultation with pharmacy patients and customers within this study is a notable limitation. A crucial next step will be to undertake collaborative work with individuals and communities to explore patients’ and customers’ perception of community pharmacy as a health asset and the most effective ways in which this could be capitalised.

## Conclusion and implications

Whilst small-scale, this study provides novel and valuable insights into the potential for community pharmacy to incorporate and contribute to asset-based approaches in their localities, and occupy a more central role in the reduction of health inequalities.
